# Oral Exposure to *Cordyceps javanica* Reduces Survival and Pupation of *Spodoptera frugiperda* and Alters Enzyme Activities and Immune-Gene Expression

**DOI:** 10.3390/insects17090933

**Published:** 2026-09-06

**Authors:** Xinghan Li, Fei Tang, Guanxi Liu, Jiaying Zhu, Wenxiu Wang

**Affiliations:** 1 Key Laboratory of Forest Disaster Warning and Control of Yunnan Province, Southwest Forestry University, Kunming 650224, China; lixinghan@swfu.edu.cn (X.L.); tang20220652005@swfu.edu.cn (F.T.); 15368258786@163.com (G.L.); jyzhu@swfu.edu.cn (J.Z.); 2 Key Laboratory for Forest Resources Conservation and Utilization in the Southwest Mountains of China, Ministry of Education, Southwest Forestry University, Kunming 650224, China

**Keywords:** entomopathogenic fungi, biological control, metamorphosis, protective and detoxification enzymes, immune defense-related genes

## Abstract

*Cordyceps javanica* (Bally) Kepler, B. Shrestha & Spatafora 2017 is an entomopathogenic fungus capable of controlling a wide range of insect pests, demonstrating substantial potential for biological control. To date, the effects of *C. javanica* on *Spodoptera frugiperda* (J.E. Smith, 1797) and the underlying mechanisms by which *S. frugiperda* responds to *C. javanica* infection remain largely unclear. In light of this, the present study conducted bioassays on *S. frugiperda* following both feeding and immersion treatments with *C. javanica*, investigated the effects on survival rate, pupation rate, and female proportion of *S. frugiperda*, evaluated the changes in protective enzyme and detoxification enzyme activities in *S. frugiperda* post-treatment, and examined the expression patterns of immune-related genes in *S. frugiperda* after treatment. The results showed that the survival rate of *S. frugiperda* fed with *C. javanica* at a concentration of 1 × 10^6^ conidia/mL was only 66%, and the pupation rate was 65.2%. In contrast, compared with the control group, the immersion treatment did not result in significant changes in the survival rate, pupation rate, or female ratio of *S. frugiperda*. In response to *C. javanica* infection, the activities of protective enzymes (POD, CAT, and SOD) and detoxification enzymes (CarE and GST) were either enhanced or inhibited. Additionally, the expression patterns of most immune-related genes were initially down-regulated and subsequently up-regulated, with the exception of *lebocin 4* and *PGRP-LB* under the feeding treatment. Notably, the feeding treatment induced a more intense defense response in *S. frugiperda*. These findings indicate that treatment with *C. javanica* affects the survival and metamorphosis of *S. frugiperda*, and that *S. frugiperda* defends against *C. javanica* infection by activating immunity. This study provides a theoretical basis for the green prevention and control of *S. frugiperda*.

## Simple Summary

*Spodoptera frugiperda* (J.E. Smith, 1797), as a polyphagous leaf-feeding pest, poses a significant threat to economically important crops. Current research on the use of entomopathogenic fungi for the control of *S. frugiperda* has primarily focused on *Beauveria bassiana* (Bals.-Criv.) Vuill. 1912, *Metarhizium anisopliae* (Metschn.) Sorokin 1883, *Metarhizium rileyi* (Farl.) Kepler, S.A. Rehner & Humber 2014, and *Cordyceps fumosorosea* (Wize) Kepler, B. Shrestha & Spatafora 2017. However, research on how *S. frugiperda* responds to entomopathogenic fungal infections is still scarce, and only *B. bassiana* has been studied in this context. In light of this, the present study investigated the effect of *Cordyceps javanica* (Bally) Kepler, B. Shrestha & Spatafora 2017, a fungus with great biocontrol potential, on *S. frugiperda*, as well as the underlying mechanisms by which *S. frugiperda* responds to *C. javanica* infection. The results showed that feeding on *C. javanica* significantly reduced both the survival rate and the pupation rate of *S. frugiperda*. The immersion treatment had no significant effect on the survival, pupation rate, or female ratio of *S. frugiperda*. The *C. javanica* treatment continuously activated protective enzymes (immersion: SOD, POD, CAT; feeding: CAT) and detoxification enzymes (immersion: CarE; feeding: CarE, GST), but suppressed SOD and POD under feeding. Immune genes under immersion showed initial down- then up-regulation. Under feeding, *gloverin* followed this pattern, whereas *attacin A* and *PGRP-LB* were persistently down-regulated, and *lebocin 4* showed the opposite trend (up then down). These findings provide a reference for the future control of *S. frugiperda* using *C. javanica*.

## 1. Introduction

*Spodoptera frugiperda* (J.E. Smith, 1797) (Lepidoptera: Noctuidae) is distributed across the Americas, Africa, and Asia [1]. The larvae of this pest cause severe damage to leaves and buds, with buds being particularly affected [2]. It also exhibits a high degree of dispersal ability and adaptability, and favors a wide range of host plants [3,4]. As a result, it causes continuous damage to food crops almost throughout the entire year, leading to significant economic losses [5]. This situation has led to the overuse or abuse of chemical insecticides, which in turn has adverse effects on both the environment and human health, and has also contributed to the development of insecticide resistance in *S. frugiperda* [6,7]. Entomopathogenic fungi have gradually become one of the sustainable control methods to effectively replace or assist chemical control and achieve efficient and green prevention and control of *S. frugiperda*.

*Cordyceps javanica* (Bally) Kepler, B. Shrestha & Spatafora 2017(Hypocreales: Cordycipitaceae) is an entomopathogenic fungus capable of controlling a wide range of insect pests. Spraying a wettable powder formulation of this fungus at 1 × 10^8^ conidia/mL caused 100% mortality in *Hebata vitis* (Göthe, 1875), while dipping *Lymantria dispar* (Linnaeus, 1758) larvae in a conidial suspension of the same concentration resulted in 90.9% mortality [8,9]. These characteristics highlight its potential as a biological control agent for use in integrated pest management (IPM) programs, particularly as a sustainable alternative or supplement to chemical insecticides. Entomopathogenic fungi typically infect insects by penetrating the cuticle through a process governed by sophisticated molecular mechanisms. Infection begins with conidial adhesion to the host cuticle, mediated by hydrophobins (HYD1/HYD2), adhesin-like proteins (Mad1), and cell wall proteins (CWP10) through hydrophobic interactions [10,11,12,13,14,15,16]. Upon germination, the fungus forms appressoria that secrete cuticle-degrading enzymes (CDEs), particularly the Pr1 family of serine proteases (especially Pr1A, which accounts for over 90% of cuticle-degrading activity) and Pr2 trypsin-like proteases, which hydrolyze the protein- and chitin-rich cuticle [17,18,19,20,21]. Overexpression of Pr1A was shown to reduce the median lethal time (LT_50_) by 25%, whereas Pr1A knockout significantly reduced fungal virulence, confirming its essential role in pathogenesis [22,23]. Concurrently, the lipid droplet protein MPL1 generates turgor pressure within appressoria to facilitate mechanical penetration [24]. Once inside the hemocoel, the fungus differentiates into yeast-like blastospores with thinner cell walls to evade immune recognition; the collagen-like protein Mcl1 further masks cell wall β-glucan to prevent hemocyte encapsulation [25,26,27,28,29,30,31]. Additionally, EPF secrete immunosuppressive secondary metabolites such as destruxins and oosporein, which inhibit antimicrobial peptide (AMP) expression and suppress prophenoloxidase activity, while high concentrations of Pr1A can directly degrade host immune proteins to accelerate mortality [23,32,33,34]. Finally, under suitable conditions, the fungus emerges from the cadaver and produces conidia to initiate new infection cycles. Besides cuticular penetration, entomopathogenic fungi can also be ingested with food and enter the digestive tract, where they interfere with nutrient absorption, growth, and survival of the insect [35,36,37,38,39]. Following ingestion, conidia of *Beauveria bassiana* (Bals.-Criv.) Vuill. 1912 persist in the midgut and hindgut of *Tribolium castaneum* (Herbst, 1797) larvae, compromising their capacity for nutrient ingestion, processing, and absorption, and subsequently resulting in host death [35]. Moreover, ingestion of *B. bassiana* conidia reduces the pupation rate of *Spodoptera litura* (Fabricius, 1775) and kills larvae of *Helicoverpa armigera* (Hübner, 1808) [37,39]. Similarly, blastospores of *Metarhizium brunneum* Petch 1935 penetrate the midgut of *Aedes aegypti* (Linnaeus, 1762), completing the infection process and leading to host death [38].

Insects rely on innate immunity to defend against pathogen infections. Upon invasion, immune cells generate reactive oxygen species (ROS), leading to oxidative stress [40]. Protective enzymes such as superoxide dismutase (SOD), catalase (CAT), and peroxidases (POD) scavenge ROS to maintain redox balance, while detoxifying enzymes including carboxylesterase (CarE) and glutathione S-transferase (GST) neutralize toxic compounds [40,41]. However, the mechanism by which *S. frugiperda* regulates these enzymes in response to fungal infection remains poorly understood.

Antimicrobial peptides (AMPs) are key components of insect innate immunity [42]. Major AMP families in Lepidoptera include cecropin (α-helical), lebocin (proline-rich), attacin and gloverin (glycine-rich) [43,44,45]. Previous studies have shown that exposure to entomopathogenic fungi can modulate AMP expression in various insect species, and the response often differs between cuticular and oral infection routes [46,47,48,49]. Peptidoglycan recognition proteins (PGRPs) are pattern recognition receptors that bind to pathogen-associated peptidoglycan molecules and activate immune signaling [50]. In *Bombyx mori* Linnaeus, 1758, *PGRP-LB* expression is upregulated in the intestine upon oral infection [51]. Currently, far less is known about the immune response of *S. frugiperda* to entomopathogenic fungal infection, with only *B. bassiana* having been documented.

In this study, we used the strain *C. javanica* BE01 to treat *S. frugiperda* larvae via two different exposure routes: immersion (simulating cuticular infection) and feeding (simulating oral infection). The specific objectives were to: (1) evaluate the effects of *C. javanica* on the survival, pupation rate, and female proportion of *S. frugiperda*; (2) compare the physiological responses (activities of protective and detoxifying enzymes) induced by the two treatment routes; and (3) examine the expression patterns of immune-related genes (*attacin A*, *lebocin 4*, *gloverin*, *cecropin*, and *PGRP-LB*) following fungal infection. By addressing these objectives, this study aims to elucidate the infection mechanisms of *C. javanica* against *S. frugiperda* and to provide insights into its potential as a biological control agent.

## 2. Material and Methods

### 2.1. Insect Rearing and Fungal Culture

Eggs of *S. frugiperda* used in this study were obtained from Henan Jiyuan Baiyun Industry Co., Ltd (Jiyuan, China). Following hatching, the larvae were reared with artificial food (Henan Jiyuan Baiyun Industry Co., Ltd., Jiyuan, China) at 28 °C ± 1 °C, 75% ± 5% relative humidity, and a 14:10 h light/dark photoperiod. Only larvae up to the second instar were used in subsequent experiments. The *C. javanica* strain BE01 (CGMCC No. 13192) was deposited in the Key Laboratory of Forest Resources Conservation and Utilization, Ministry of Education, Southwest China (Kunming, China). The BE01 strain was grown on Sabouraud dextrose agar with yeast extract (SDAY) containing 1% tryptone, 1% yeast extract, 4% glucose, and 2% agar, in sealed Petri dishes at 25 °C. Conidia were harvested according to Wang [52] and then diluted with 0.1% Tween-80 to prepare spore suspensions of 1 × 10^4^ and 1 × 10^6^ conidia/mL. A 0.1% Tween-80 solution served as the control for subsequent bioassays and RT-qPCR analysis.

### 2.2. Bioassay of Spodoptera frugiperda After Treatment with Cordyceps javanica BE01

#### 2.2.1. Survival Rate, Pupation Rate and Female Rate

Immersion treatment: The 2nd instar larvae were immersed for 15 s in spore suspensions of 1 × 10^4^ or 1 × 10^6^ conidia/mL, then left to crawl on sterile filter paper until their body surfaces were dry. Each larva was kept in a separate insect box with an artificial diet cube (0.5 cm^3^), which was renewed every 12 h. The diet was a commercial product obtained from Henan Jiyuan Baiyun Industry Co., Ltd (Jiyuan, China). Its exact composition is proprietary to the manufacturer and cannot be disclosed. All diet cubes used in this study were cut from the same bulk purchase to avoid batch-to-batch variation and were stored according to the supplier’s instructions until use. Larvae treated with 0.1% Tween-80 solution were used as controls. The boxes were then placed in a constant-temperature incubator under the conditions specified in Section 2.1. Feeding treatment: The artificial diet was cut into 0.5 cm^3^ cubes. Each cube was completely immersed in spore suspensions at concentrations of 1 × 10^4^ or 1 × 10^6^ conidia/mL for 30 s. After removal, the cubes were placed on sterile filter paper and allowed to air-dry naturally until the surface spore solution had dried. Each insect box contained one second-instar larva and one piece of contaminated diet (treated with the spore suspension only at the beginning, followed by sterile diet every 12 h thereafter). Artificial diet treated with 0.1% Tween-80 aqueous solution was provided to the control group. Mortality was recorded daily over a 15-day period after immersion or feeding treatment, respectively. To confirm that mortality was caused by fungal infection, dead larvae from the fungal treatment groups were surface-sterilized with 75% ethanol, rinsed three times with sterile distilled water, and placed on moist sterile filter paper at 25 ± 1 °C for 2–3 days to observe fungal outgrowth (mycosis). Fungal mycelia were observed emerging from cadavers of the fungal-treated groups under a light microscope, whereas no such growth was observed in the control group. For the feeding treatment, cadavers were subjected to the same surface-sterilization and incubation procedure. However, outgrowth of fungal mycelia on the cadaver surface was not consistently observed, consistent with the oral route of infection. After 15 days, the surviving larvae were reared further, and the numbers of pupae and female adults were counted. For the immersion treatment, each of the 1 × 10^4^ conidia/mL group, 1 × 10^6^ conidia/mL group, and control group contained 100 larvae, totaling 300 larvae; the same was applied to the feeding treatment.

#### 2.2.2. Determination of Enzyme Activity

For enzyme activity determination, the 2nd instar larvae were treated as described in Section 2.2.1, but using 1 × 10^6^ conidia/mL *C. javanica* for both immersion and feeding treatments, with 0.1% Tween-80 as the control. At 12, 24, 36, 48, and 60 h post-treatment, approximately 15–20 larvae per replicate from each treatment group were pooled, ground under liquid nitrogen, and 0.1 g of the homogenate was weighed for each sample following the enzyme activity assay kit manufacturer’s protocol. Subsequently, 1 mL of pre-cooled extraction solution from the kit was added, maintaining a larval mass-to-extract volume ratio of 1:10. After being fully ground and mixed in an ice bath, the homogenates were centrifuged at 8000 rpm for 10 min at 4 °C. The activities of superoxide dismutase (SOD), catalase (CAT), peroxidase (POD), carboxylesterase (CarE), and glutathione S-transferase (GST) were then determined following the protocols provided by the manufacturer (SOD kit: BC0175; POD kit: BC0095; CAT kit: BC0205; CarE kit: BC0845; GST kit: BC0355, Solarbio Co., Ltd., Beijing, China). All assays were performed in 96-well plates (96-well UV-transparent plates for CAT and GST) using a microplate reader. Enzyme activities were expressed as units per gram of fresh weight (U/g).

For SOD (absorbance at 560 nm), the inhibition percentage was calculated as: Inhibition (%) = (ΔA_blank − ΔA_sample)/ΔA_blank × 100, where ΔA_blank = A1_blank − A2_blank (difference between blank tubes 1 and 2), and ΔA_sample = A_sample − A_control (difference between sample tube and its paired control). The reaction was incubated at 37 °C for 30 min. For the 96-well plate format, SOD activity (U/g) was calculated as: SOD (U/g) = 10 × [Inhibition/(1 − Inhibition)]/W × F, where W is the sample weight (g), and F is the dilution factor (if the sample was diluted before the assay; otherwise F = 1). The constant 10 is derived from the reaction volume (0.2 mL), sample volume (0.02 mL), total extraction volume (1 mL), and the unit-definition system (1 mL reaction system). This formula is equally valid for 96-well plate measurements as specified by the manufacturer.

For POD (absorbance at 470 nm), the absorbance change ΔA = A2 − A1 was recorded over 1 min (from 30 s to 1 min 30 s after initiation). POD activity (U/g) in the 96-well plate format was calculated as:POD (U/g) = 9800 × ΔA/W × F, where W and F are as above. The constant 9800 incorporates the total reaction volume (0.245 mL), sample volume (0.005 mL), reaction time (1 min), and the unit-definition threshold of 0.005 absorbance change per minute per mL in a 96-well plate (as specified in the kit manual).

For CAT (absorbance at 240 nm, UV-plate), the decrease in absorbance ΔA = A1 − A2 was monitored over 1 min (from 5 s to 1 min 5 s). CAT activity (U/g) in the 96-well UV-plate format was calculated as: CAT (U/g) = 764.5 × ΔA/W × F, where W and F are as above. The constant 764.5 is derived from the total reaction volume (2 × 10^−4^ L), the molar extinction coefficient of H_2_O_2_ (ε = 43.6 L·mol^−1^·cm^−1^), the optical path length of the 96-well UV plate (d = 0.6 cm), the sample volume (0.01 mL), the reaction time (1 min), and the unit conversion factor (10^6^ μmol·mol^−1^).

For CarE (absorbance at 450 nm), the absorbance was measured at 10 s (A1) and at 5 min 10 s (A2) for both sample and blank tubes. The net absorbance change for the sample was ΔA_sample = A2_sample − A1_sample, and for the blank ΔA_blank = A2_blank − A1_blank. The reaction was performed at 37 °C for 5 min. CarE activity (U/g) in the 96-well plate format was calculated as: CarE (U/g) = 8 × (ΔA_sample − ΔA_blank)/W × F, where W and F are as above. The constant 8 is derived from the total reaction volume (0.2 mL), the unit-definition threshold of 0.5 absorbance change per minute per mL in a 96-well plate, the sample volume (0.01 mL), and the reaction time (5 min).

For GST (absorbance at 340 nm, UV-plate), the absorbance was recorded at 10 s (A1) and 5 min 10 s (A2) for both sample and blank. The net change ΔA = (A2_sample − A1_sample) − (A2_blank − A1_blank) was used. GST activity (U/g) in the 96-well UV-plate format was calculated as: GST (U/g) = 0.38 × ΔA/W × F, where W and F are as above. The constant 0.38 incorporates the molar extinction coefficient of the product (ε = 9.6 × 10^3^ L·mol^−1^·cm^−1^), the optical path (d = 0.6 cm), the total reaction volume (2.2 × 10^−4^ L), the sample volume (0.02 mL), the reaction time (5 min), and the unit conversion factor (10^6^ μmol·mol^−1^).

For SOD, CarE, and GST assays, a reagent blank (containing all reagents but no sample) was included and its absorbance was subtracted from the corresponding sample readings. For POD and CAT, the manufacturer’s protocols do not include blank tubes, and enzyme activities were calculated directly from the absorbance changes of sample readings according to the kit instructions. Reagent blanks (when used) were not plotted as independent data points or included in statistical analyses. The experiment was repeated three times.

### 2.3. RNA Extraction, Complementary DNA (cDNA) Synthesis and RT-qPCR Analysis

For RNA extraction, the immersion and feeding treatments of 2nd instar *S. frugiperda* larvae were conducted according to Section 2.2.1, except that the treatment groups received a *C. javanica* suspension of 1 × 10^6^ conidia/mL, and the control group received 0.1% Tween-80 aqueous solution. At 12, 24, 36, 48, and 60 h after treatment, RNA was extracted and cDNA was synthesized from each sample (Three whole larvae frozen in liquid nitrogen after treatment) following the protocol of Wang [53]. Gene-specific primers targeting the cDNA sequences of immune-related genes were then designed (Table S1). Elongation factor 1 alpha (EF1α) served as an internal reference. RT-qPCR was carried out in a 20 μL reaction volume using SYBR^®^ Premix Ex Taq II (Takara Biomedical Technology Co., Ltd., Beijing, China). RT-qPCR was performed under the following conditions: initial denaturation at 95 °C for 1 min, then 45 cycles of denaturation at 95 °C for 15 s and annealing at 60 °C for 30 s. The experiment was repeated three times.

### 2.4. Data Analysis

Survival curves were plotted using GraphPad Prism 9.5 (GraphPad Software, Inc., San Diego, CA, USA). Cumulative survival rates were calculated using the Kaplan–Meier method. The log-rank test was employed to compare survival rates between the 0.1% Tween-80 aqueous solution group and the groups treated with different concentrations of the conidial suspension. The chi-square test in SPSS 27.0.1.0 (SPSS Inc., Chicago, IL, USA) was used to analyze the significance of differences in pupation rate and female ratio following treatment with 0.1% Tween-80 aqueous solution versus different conidial suspension concentrations. Differences in enzyme activity or immune-related gene expression between the 0.1% Tween-80 aqueous solution group and the conidial suspension-treated group at the same time point were analyzed using Student’s *t*-test in SPSS.

## 3. Results

### 3.1. Effects of Cordyceps javanica on the Survival and Growth of Spodoptera frugiperda

**Figure 1:**
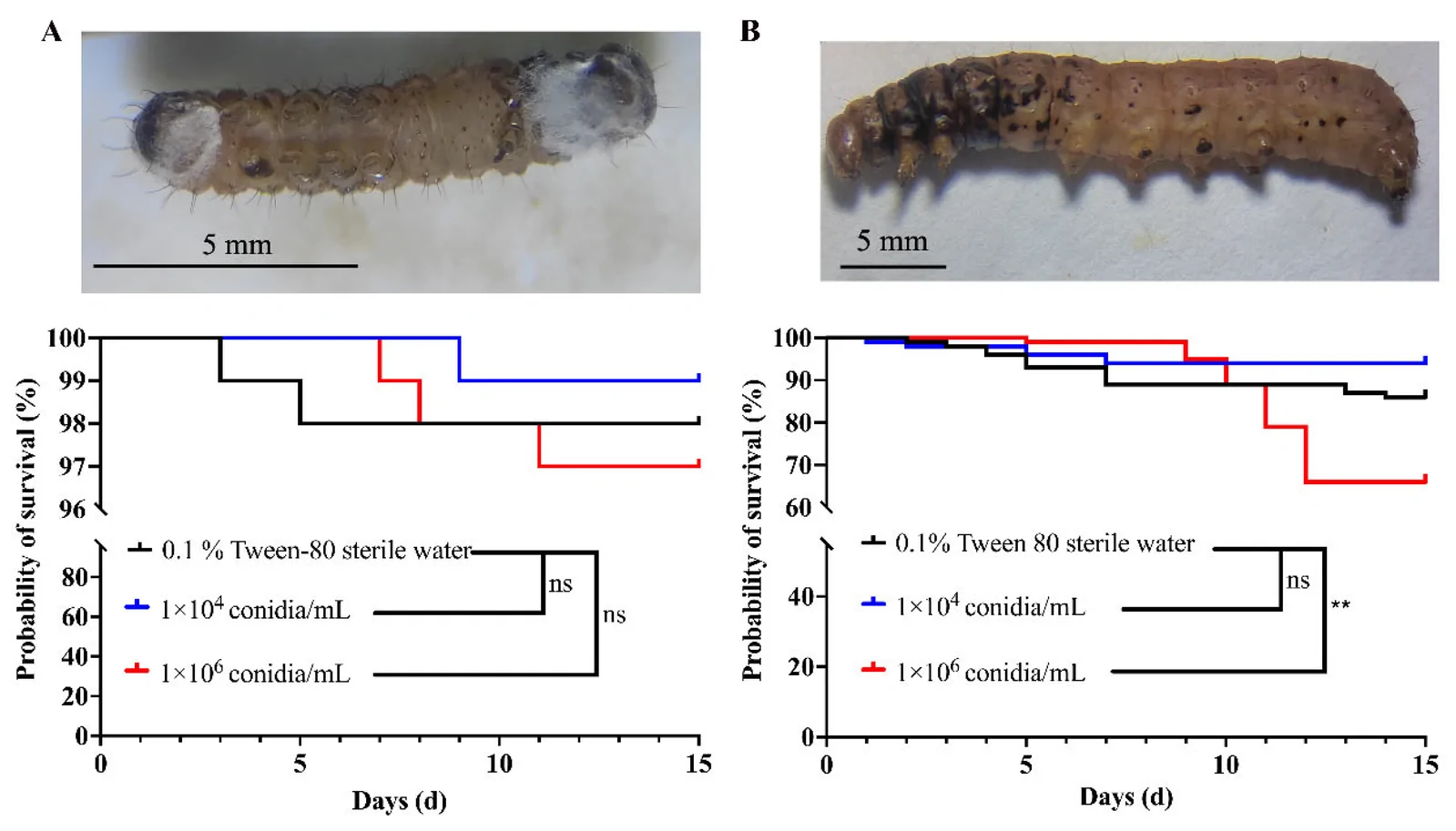
Effects of different concentrations (1 × 10^4^ and 1 × 10^6^ conidia/mL) of *Cordyceps javanica* on the survival of *Spodoptera frugiperda* larvae over a 15-day observation period. (**A**) Immersion treatment; (**B**) feeding treatment. The abscissa represents days post-treatment, and the ordinate represents the larval survival rate (%). Statistical significance was determined using the chi-square test. ns: no significant difference; **: significant difference at *p* < 0.01. The photomicrographs (taken under a stereomicroscope) show representative dead larvae from the corresponding treatments, illustrating typical pathological features including mummification and softening.

Immersion treatment with *C. javanica* exhibited virtually no lethal effect on *S. frugiperda* (Figure 1A, Table S2). There was no significant difference in the survival rate of *S. frugiperda* larvae treated with different concentrations of *C. javanica* compared with the control (χ^2^ = 1.010, df = 2, *p* = 0.603, *n* = 300). The mortality rate at the highest concentration (10^6^ conidia/mL) was only 3%. Furthermore, the pupation rates of *S. frugiperda* following treatment with 0.1% Tween-80 sterile water, 1 × 10^4^ conidia/mL, and 1 × 10^6^ conidia/mL suspensions were 93.9%, 93.9%, and 85.6%, respectively, with no significant difference among groups (χ^2^ = 5.610, df = 2, *p* = 0.060, *n* = 294). The pupation rates of the two different concentration treatments were not significantly different from that of the control (Table 1). Similarly, no significant difference was observed in the female emergence rate among the different concentration treatments and the 0.1% Tween-80 sterile water control (χ^2^ = 1.442, df = 2, *p* = 0.486, *n* = 268). The female rates of *S. frugiperda* treated with the two different concentrations did not differ significantly from that of the control (Table 1).

Feeding with *C. javanica* had a lethal effect on *S. frugiperda* (Figure 1B, Table S3). There was a significant difference in the survival rate of *S. frugiperda* larvae among different treatments (χ^2^ = 25.420, df = 2, *p* < 0.0001, *n* = 300). Among the tested concentrations, the lowest survival rate (66%) was observed at 12 days post-treatment following feeding with the 1 × 10^6^ conidia/mL suspension, which was significantly lower than that of the control (χ^2^ = 10.965, df = 1, *p* < 0.001). Similarly, the pupation rate of larvae fed with the 1 × 10^6^ conidia/mL suspension (65.2%) was significantly lower than that of the control (96.5%), whereas larvae treated with 1 × 10^4^ conidia/mL showed a pupation rate of 90.4% (χ^2^ = 32.793, df = 2, *p* < 0.001). Detailed statistical results are presented in Table 2. Meanwhile, no significant difference was observed in the female emergence rate after feeding with different concentrations of the spore suspension compared with the 0.1% Tween-80 sterile water control (χ^2^ = 0.243, df = 2, *p* = 0.886, *n* = 211).

### 3.2. Effects of Cordyceps javanica on Protective Enzymes and Detoxification Enzymes of Spodoptera frugiperda

**Figure 2:**
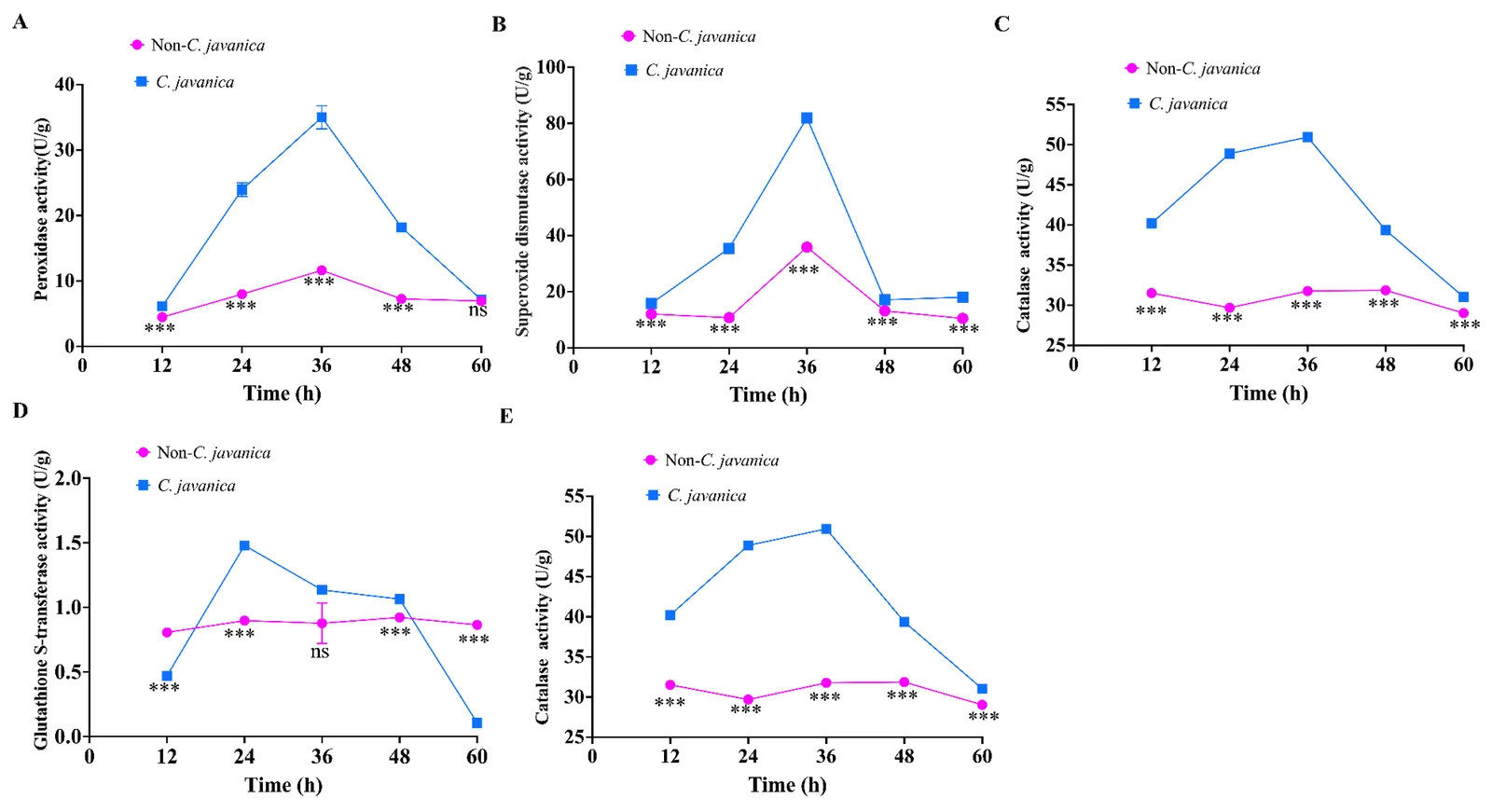
Effects of immersion treatment with *Cordyceps javanica* on the activities of protective and detoxification enzymes in *Spodoptera frugiperda* larvae. (**A**) Peroxidase (POD) activity; (**B**) Superoxide dismutase (SOD) activity; (**C**) Catalase (CAT) activity; (**D**) Carboxylesterase (CarE) activity; (**E**) Glutathione S-transferase (GST) activity. Non-*C. javanica*: control larvae immersed in sterile distilled water with 0.1% Tween-80 without conidia; *C. javanica*: larvae immersed in *C. javanica* conidial suspension at 1 × 10^6^ conidia/mL. The abscissa indicates time after immersion treatment (hours); the ordinate indicates enzyme activity (U/g). Data are presented as mean ± SE. Student’s *t*-test was used for significance analysis between treatment groups and the control at the same time point for each enzyme. ns: no significant difference; ***: significant difference at *p* < 0.001.

Immersion treatment with *C. javanica* BE01 significantly modulated the activities of protective and detoxification enzymes in *S. frugiperda*. The activities of peroxidases (POD), superoxide dismutase (SOD), and catalase (CAT) were generally higher in the immersion-treated group than in the control group from 12 to 60 h post-treatment, except for POD at 60 h, which showed no significant difference from the control (SOD: 12 h: *t* = 12.964, df = 12, *p* < 0.001; 24 h: *t* = 46.736, df = 7.222, *p* < 0.001; 36 h: *t* = 74.581, df = 10, *p*< 0.001; 48 h: *t* = 8.673, df = 12, *p* < 0.001; 60 h: *t* = 32.938, df = 12, *p* < 0.001; POD: 12 h: *t*= 5.654, df = 14, *p* < 0.001; 24 h: *t* = 14.521, df = 12, *p* < 0.001; 36 h: *t* = 13.133, df = 6.416, *p* < 0.001; 48 h: *t* = 15.457, df = 14, *p* < 0.001; 60 h: *t* = 0.463, df = 14, *p* > 0.05; CAT: 12 h: *t* = 73.698, df = 13, *p* < 0.001; 24 h: *t* = 82.284, df = 14, *p* < 0.001; 36 h: *t* = 75.619, df = 12, *p* < 0.001; 48 h: *t* = 21.868, df = 12, *p* < 0.001; 60 h: *t* = 6.708, df = 7.564, *p* < 0.001) (Figure 2A–C). All three protective enzymes showed an initial increase followed by a decrease, peaking at 36 h (Figure 2A–C).

For detoxification enzymes, carboxylesterase (CarE) and glutathione S-transferase (GST) activities reached their highest levels at 24 h post-treatment (Figure 2D,E). CarE activity was significantly higher than that of the control at all time points except 12 h (12 h: *t* = 8.576, df = 12, *p* < 0.001; 24 h: *t* = 36.143, df = 6.640, *p* < 0.001; 36 h: *t* = 40.097, df = 10, *p* < 0.001; 48 h: *t* = 12.631, df = 11, *p* < 0.001; 60 h: *t* = 34.640, df = 12, *p* < 0.001). GST activity was significantly higher than the control at 24 h and 48 h, but not at 12, 36, or 60 h (12 h: *t* = 16.648, df = 9.434, *p* < 0.001; 24 h: *t* = 62.519, df = 12, *p* < 0.001; 36 h: *t* = 1.646, df = 10, *p* > 0.05; 48 h: *t* = 13.293, df = 12, *p* < 0.001; 60 h: *t* = 114.316, df = 6.230, *p* < 0.001).

**Figure 3:**
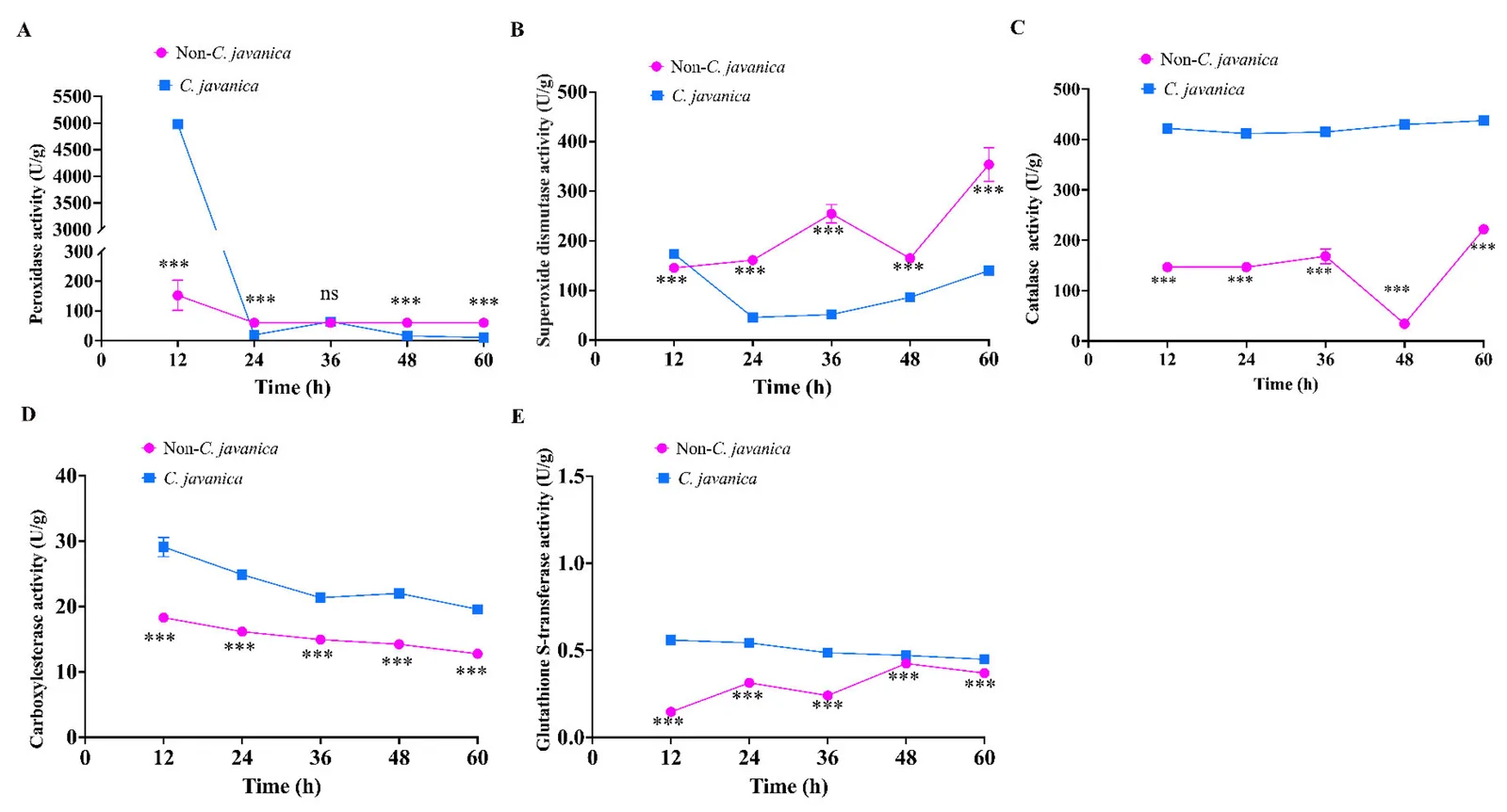
Effects of feeding with *Cordyceps javanica*-treated diet on the activities of protective and detoxification enzymes in *Spodoptera frugiperda* larvae. (**A**) Peroxidase (POD) activity; (**B**) superoxide dismutase (SOD) activity; (**C**) catalase (CAT) activity; (**D**) carboxylesterase (CarE) activity; (**E**) glutathione S-transferase (GST) activity. Non-*C. javanica*: larvae fed with artificial diet without *C. javanica* conidia (treated with sterile distilled water containing 0.1% Tween-80 only); *C. javanica*: larvae fed with artificial diet with 1 × 10^6^ conidia/mL *C. javanica.* The abscissa indicates time after feeding treatment (hours); the ordinate indicates enzyme activity (U/g). Data are presented as mean ± SE. Student’s *t*-test was used for significance analysis between treatment groups and the control at the same time point for each enzyme. ns: no significant difference; ***: significant difference at *p* < 0.001.

Feeding with *C. javanica* BE01 at different time points also induced changes in the activities of protective and detoxification enzymes in *S. frugiperda*. The activities of POD and SOD were higher than those of the control only at 12 h after feeding with BE01, and were significantly lower than those of the control at the other time points (POD: 12 h: t = 80.361, df = 12, *p* < 0.001; 24 h: t = 11.722, df = 9.956, *p* < 0.001; 36 h: t = 0.601, df = 8.606, *p* > 0.05; 48 h: t = 12.572, df = 9.938, *p* < 0.001; 60 h: t = 13.751, df = 8.605, *p* < 0.001; SOD: 12 h: t = 16.786, df = 16, *p* < 0.001; 24 h: t = 12.983, df = 8.067, *p* < 0.001; 36 h: t = 10.864, df = 8.008, *p* < 0.001; 48 h: t = 7.923, df = 8.034, *p* < 0.001; 60 h: t = 6.245, df = 8.002, *p* < 0.001) (Figure 3A,B). Notably, POD activity at 12 h after feeding was extremely high, nearly 25 times that of the control (Figure 3A). In addition, the activities of CAT, CarE, and GST were significantly higher than those of the control group at 12–60 h (CAT: 12 h: t = 82.810, df = 8, *p* < 0.001; 24 h: t = 92.089, df = 8, *p* < 0.001; 36 h: t = 16.513, df = 8.592, *p* < 0.001; 48 h: t = 63.940, df = 15, *p* < 0.001; 60 h: t = 53.256, df = 8.007, *p* < 0.001; CarE: 12 h: t = 7.329, df = 8.001, *p* < 0.001; 24 h: t = 282.208, df = 10.266, *p* < 0.001; 36 h: t = 10.380, df = 8.001, *p* < 0.001; 48 h: t = 89.080, df = 16, *p* < 0.001; 60 h: t = 256.139, df = 8.727, *p* < 0.001; GST: 12 h: t = 16.775, df = 16, *p* < 0.001; 24 h: t = 12.250, df = 16, *p* < 0.001; 36 h: t = 25.374, df = 16, *p* < 0.001; 48 h: t = 7.462, df = 9.171, *p* < 0.001; 60 h: t = 18.539, df = 16, *p* < 0.001) (Figure 3C–E).

### 3.3. Effects of Cordyceps javanica on Expression Patterns of Immune-Related Genes in Spodoptera frugiperda

**Figure 4:**
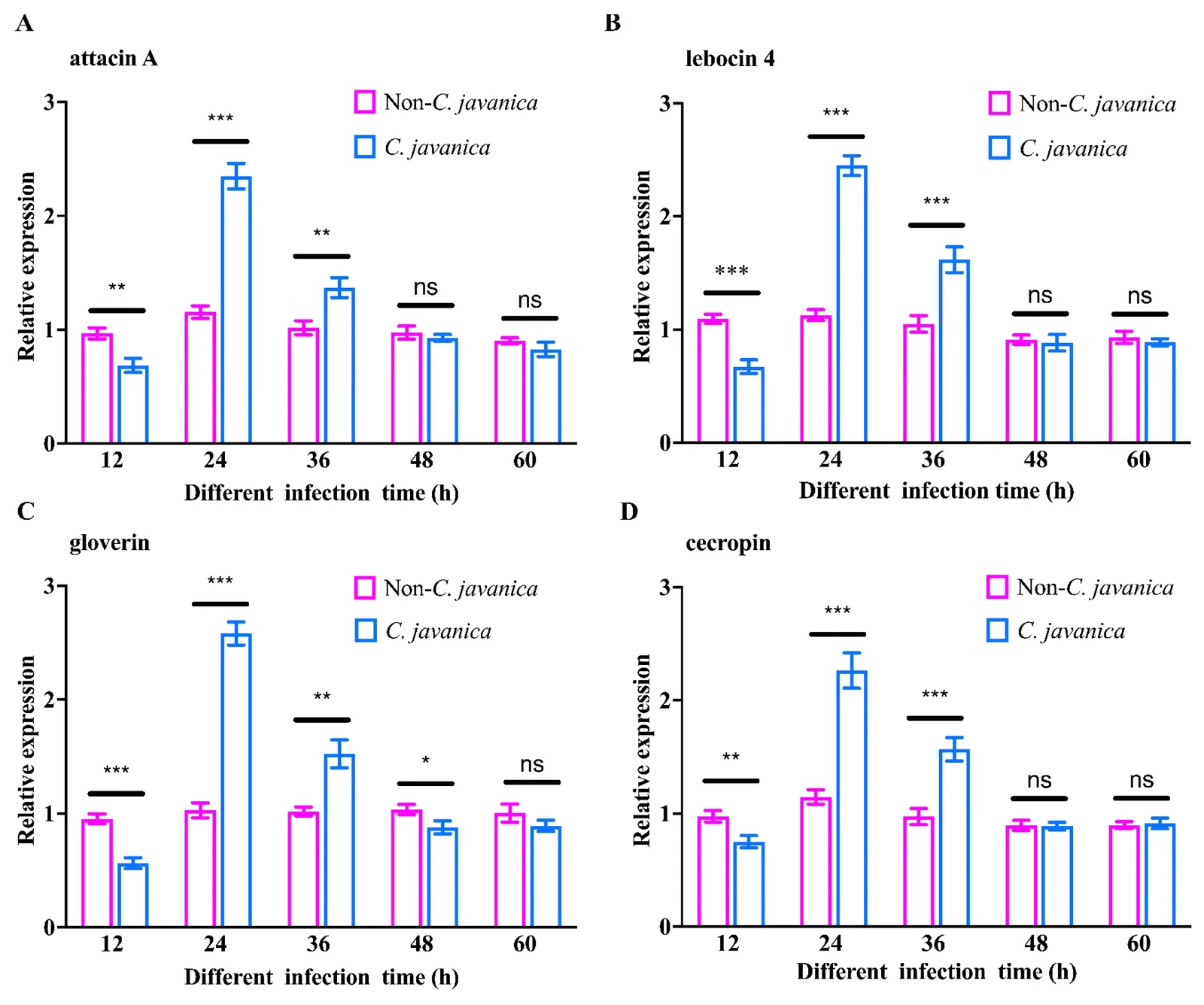
Expression patterns of immune-related genes in *Spodoptera frugiperda* larvae at different time points after *Cordyceps javanica* infection. (**A**) *attacin A*; (**B**) *lebocin 4*; (**C**) *gloverin*; (**D**) *cecropin*. Non-*C. javanica*: control larvae immersed in sterile distilled water containing 0.1% Tween-80 without conidia; *C. javanica*: larvae immersed in *C. javanica* conidial suspension at 1 × 10^6^ conidia/mL. The abscissa indicates time after treatment (hours); the ordinate indicates relative expression level (fold change). Relative expression levels were calculated using the 2^−ΔΔCt^ method, with Elongation factor 1 alpha (*EF1α*) as the internal reference gene. Data are presented as mean ± SE. Student’s *t*-test was used for significance analysis between the treatment group and the control for the same gene at the same time point. ns: no significant difference; *: *p* < 0.05; **: *p* < 0.01; ***: *p* < 0.001.

The expression of the immune-related genes *attacin A*, *lebocin 4*, *gloverin*, and *cecropin* in *S. frugiperda* was induced by *C. javanica* immersion. The expression level of *attacin A* was significantly lower than that of the control at 12 h post-immersion, but significantly higher than the control at 24 h and 36 h post-immersion (Figure 4A). At 48 h and 60 h post-immersion, no significant difference was observed compared with the control (12 h: t = 3.550, df = 22, *p* < 0.01; 24 h: t = 9.512, df = 16.058, *p* < 0.001; 36 h: t = 3.324, df = 22, *p* < 0.01; 48 h: t = 0.693, df = 22, *p* > 0.05; 60 h: t = 1.567, df = 11.079, *p* > 0.05) (Figure 4A). The expression of *lebocin 4* was significantly lower than that of the control at 12 h post-immersion, but significantly higher than the control at 24 h and 36 h post-immersion. No significant difference was observed at 48 h or 60 h post-immersion (12 h: t = 5.806, df = 19.375, *p* < 0.001; 24 h: t = 13.143, df = 17.224, *p* < 0.001; 36 h: t = 4.199, df = 18.899, *p* < 0.001; 48 h: t = 0.320, df = 17.215, *p* > 0.05; 60 h: t = 0.692, df = 22, *p* > 0.05) (Figure 4B). The expression of *gloverin* was significantly lower than that of the control at 12 h post-immersion, but significantly higher than the control at 24 h, 36 h, and 48 h post-immersion. No significant difference was observed at 60 h post-immersion (12 h: t = 6.024, df = 22, *p* < 0.001; 24 h: t = 12.875, df = 18.900, *p* < 0.001; 36 h: t = 3.943, df = 13.160, *p* < 0.01; 48 h: t = 2.195, df = 22, *p* < 0.05; 60 h: t = 1.213, df = 18.234, *p* > 0.05) (Figure 4C). The expression level of *cecropin* was significantly lower than that of the control at 12 h post-immersion, but significantly higher than the control at 24 h and 36 h post-immersion. No significant difference was observed at 48 h or 60 h post-immersion (12 h: t = 3.029, df = 22, *p* < 0.01; 24 h: t = 6.600, df = 14.613, *p* < 0.001; 36 h: t = 4.732, df = 22, *p* < 0.001; 48 h: t = 0.113, df = 22, *p* > 0.05; 60 h: t = 0.289, df = 22, *p* > 0.05) (Figure 4D).

**Figure 5:**
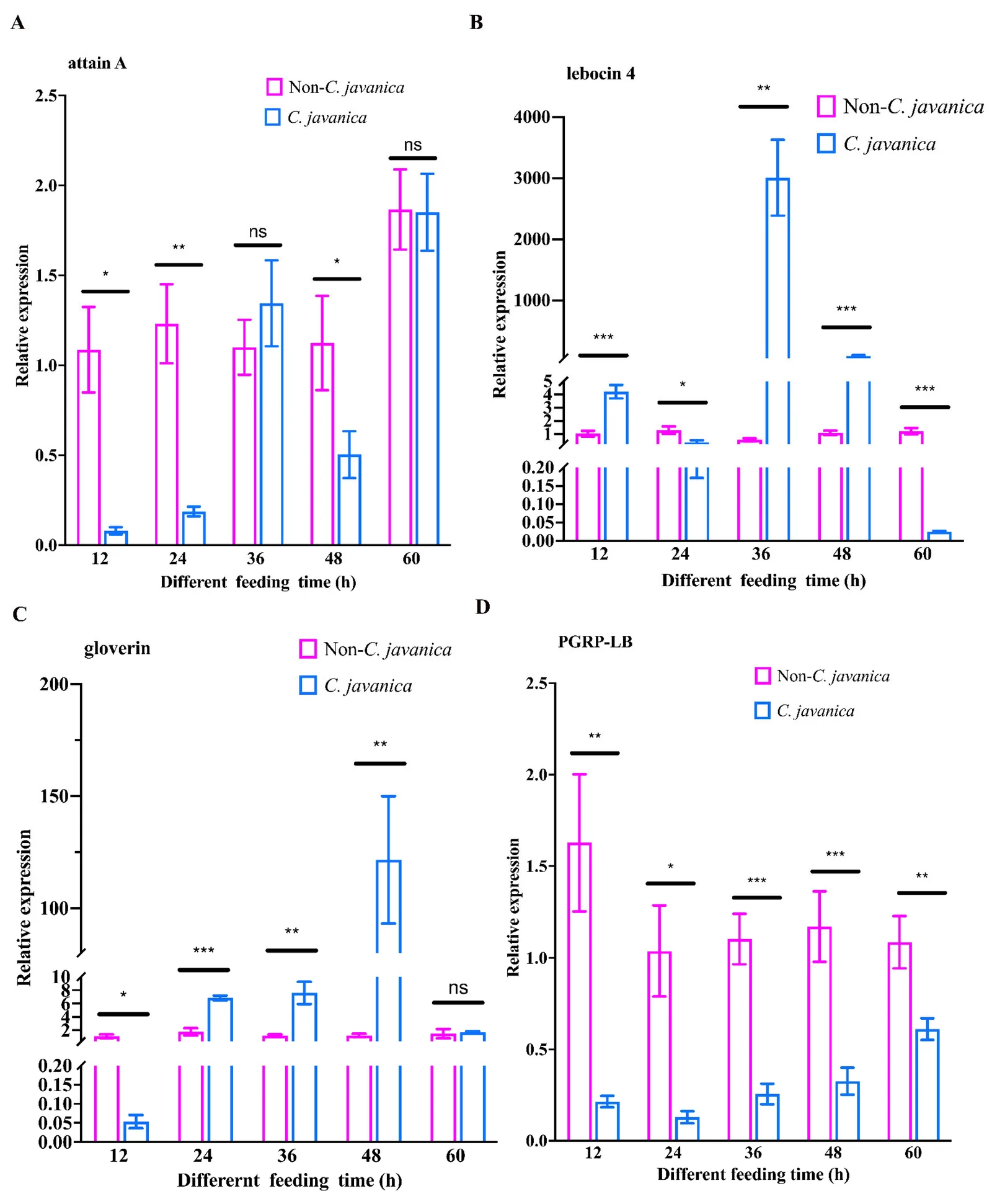
Expression patterns of immune-related genes in *Spodoptera frugiperda* larvae at different time points after feeding with *Cordyceps javanica*-treated diet. (**A**) *attacin A*; (**B**) *lebocin 4*; (**C**) *gloverin*; (**D**) *PGRP-LB*. Non-*C. javanica*: larvae fed with artificial diet without *C. javanica* conidia (treated with sterile distilled water containing 0.1% Tween-80 only); *C. javanica*: larvae fed with artificial diet with 1 × 10^6^ conidia/mL *C. javanica*. The abscissa indicates time after treatment (hours); the ordinate indicates relative expression level (fold change). Relative expression levels were calculated using the 2^−ΔΔCt^ method, with Elongation factor 1 alpha (*EF1α*) as the internal reference gene. Data are presented as mean ± SE. Student’s *t*-test was used for significance analysis between the treatment group and the control for the same gene at the same time point. ns: no significant difference; *: *p* < 0.05; **: *p* < 0.01; ***: *p* < 0.001.

Feeding with *C. javanica* affected the expression of immune-related genes in *S. frugiperda*. The expression of *attacin A* was significantly inhibited at 12 h, 24 h, and 48 h after feeding, while no significant difference was observed at the other time points compared with the control (12 h: t = 4.222, df = 3.045, *p* < 0.05; 24 h: t = 4.715, df = 9.266, *p* < 0.01; 36 h: t = 0.877, df = 21, *p* > 0.05; 48 h: t = 2.387, df = 15, *p* < 0.05) (Figure 5A). The expression of *lebocin 4* was up-regulated at 12 h, 36 h, and 48 h after feeding, but was inhibited at 24 h and 60 h after feeding (12 h: t = 5.788, df = 10.833, *p* < 0.001; 24 h: t = 2.961, df = 14, *p* < 0.05; 36 h: t = 4.640, df = 7.000, *p* < 0.01; 48 h: t = 5.575, df = 9.002, *p* < 0.001; 60 h: t = 4.911, df = 9.001, *p* < 0.001) (Figure 5B). The expression of *gloverin* was increased at 24–48 h after feeding, but was inhibited at 12 h after feeding. No significant difference was observed between the treatment and the control at 60 h after feeding (12 h: t = 3.916, df = 3.026, *p* < 0.05; 24 h: t = 8.112, df = 19, *p* < 0.001; 36 h: t = 3.807, df = 7.222, *p* < 0.01; 48 h: t = 4.245, df = 9.001, *p* < 0.01; 60 h: t = 0.339, df = 8.329, *p* > 0.05) (Figure 5C). The expression of *PGRP-LB* was inhibited from 12 to 60 h after feeding (12 h: t = 5.291, df = 6.097, *p* < 0.01; 24 h: t = 3.213, df = 6.245, *p* < 0.05; 36 h: t = 6.074, df = 17, *p* < 0.001; 48 h: t = 4.643, df = 9.733, *p* < 0.001; 60 h: t = 3.846, df = 16, *p* < 0.01) (Figure 5D).

## 4. Discussion

The effects of different treatments of *Cordyceps javanica* on the survival and pupation rates of *Spodoptera frugiperda* were markedly different. Feeding treatment resulted in a significant decrease in both survival and pupation rates compared with the control. In contrast, the immersion treatment had no significant effect on either parameter—a result obtained using a fungal strain originally isolated from *Hyphantria cunea* (Drury, 1773)—which may be attributable to the host specificity of entomopathogenic fungi during cuticular infection [54]. The activities of the protective enzymes (POD, SOD, and CAT) and detoxification enzymes (CarE and GST) in *S. frugiperda* changed between 12 and 60 h after infection or feeding with *C. javanica*. Concurrently, the expression of immune-related genes in *S. frugiperda* was either upregulated or inhibited. The above results indicate that feeding with *C. javanica* has a lethal effect on *S. frugiperda* and can influence its metamorphosis development. Although *S. frugiperda* defends against pathogen infection through protective enzymes, detoxification enzymes, and immune-related proteins, *C. javanica* may facilitate its own infection and kill the host by suppressing the expression of certain immune-related genes.

*C. javanica* affects the survival and metamorphosis of *S. frugiperda*. However, the survival rate of *S. frugiperda* did not decrease significantly after immersion treatment with *C. javanica*, which may be attributed to the fact that the fungal strain was isolated from *H. cunea*. A considerable proportion of entomopathogenic fungi are highly specialized to their hosts, and even their ability to infect closely related hosts is very poor [55]. Therefore, the *C. javanica* strain isolated from *H. cunea* exhibits low infectivity toward *S. frugiperda*. In addition to cuticular invasion, entomopathogenic fungi can infect the host by entering the digestive tract through contaminated food [38]. Feeding with *C. javanica* at a concentration of 1 × 10^6^ conidia/mL significantly reduced the survival rate of *S. frugiperda*, which is consistent with the significant decrease in survival rates observed in *S. litura* larvae fed with *B. bassiana* and in *S. frugiperda* larvae fed with *Metarhizium anisopliae* (Metschn.) Sorokin 1883 [37,56]. In addition, feeding *S. frugiperda* larvae with a high concentration of *C. javanica* significantly reduced the pupation rate by 12–31.3% compared with the control. This effect was similar to that observed in *S. litura* larvae fed with *B. bassiana*, whereas the female rate remained unaffected [37]. In summary, feeding with *C. javanica* has a stronger effect on *S. frugiperda* than immersion. In nature, entomopathogenic fungi of the order Hypocreales, including *Cordyceps* species, primarily infect their insect hosts through direct penetration of the cuticle [57]. Conidia that contact the host cuticle germinate and penetrate the integument using mechanical pressure and hydrolytic enzymes such as proteases and chitinases [58,59]. However, ingestion (*per os*) represents an alternative infection route that has been documented for some entomopathogenic fungi [60], with ingested conidia potentially germinating in the midgut, disrupting intestinal function, or releasing toxins that impair host physiology [61]. *C. javanica* affected both survival and metamorphosis of *S. frugiperda*, but immersion treatment did not cause significant mortality, possibly because the strain was isolated from *H. cunea*. Host specificity of entomopathogenic fungi largely depends on cuticular recognition and penetration efficiency, as different insect cuticular hydrocarbon profiles serve as recognition signals [62]. The *C. javanica* BE01 strain causes >73% mortality in *H. cunea* [52], but may poorly recognize *S. frugiperda* cuticle. In contrast, the feeding route bypasses this barrier, allowing conidia to directly interact with the midgut epithelium and exert sublethal effects (e.g., impaired pupation) even when cuticular infection is inefficient. In the present study, both immersion (cuticular contact) and feeding (ingestion) treatments resulted in significant physiological responses in *S. frugiperda*, suggesting that *C. javanica* may infect the host through multiple entry gates, as reported for other hypocrealean fungi such as *Beauveria bassiana* [60]. The feeding route, which does not require breaching the cuticular barrier, allows conidia to directly interact with the midgut epithelium, thereby disrupting digestive and absorptive functions and potentially weakening the host more rapidly than cuticular infection alone. Furthermore, fungal proliferation in the insect gut directly contributes to host mortality and simultaneously compromises intestinal integrity, enabling opportunistic pathogens that have overgrown following fungal infection to breach the gut barrier and translocate into the hemocoel, where they establish systemic infection and accelerate host death [63,64,65,66]. This explains the different outcomes between the two inoculation routes.

The activities of protective and detoxification enzymes in *S. frugiperda* were differentially modulated following *C. javanica* infection, suggesting their potential involvement in the host defense response. Insects alleviate oxidative stress via their antioxidant defense systems and counteract ROS damage resulting from normal metabolism or exposure to foreign organisms [40]. Protective enzymes (SOD, POD, and CAT) and detoxification enzymes (CarE and GST) are important components of this system [67,68,69,70,71]. When insects are infected by fungi, ROS accumulate in large quantities, disrupting oxygen homeostasis and activating these protective enzymes [72]. Under adverse conditions, SOD catalyzes the conversion of the superoxide radical (O_2_^−^) into hydrogen peroxide and molecular oxygen, while POD and CAT catalyze the decomposition of hydrogen peroxide into water, thereby preventing hydrogen peroxide accumulation within cells and maintaining a low level of ROS in insects [73,74,75,76]. Our study found that the activities of the protective enzymes POD, SOD, and CAT in *S. frugiperda* increased during the early stage following *C. javanica* immersion treatment, reached their maximum at 36 h, and then gradually decreased. A similar trend in protective enzyme activities was also observed in *Phyllotreta striolata* (Fabricius, 1801) and *S. frugiperda* after treatment with *M. anisopliae* and *B. bassiana*, respectively [40,77]. This may be because the activities of protective enzymes increase to resist fungal infection. Once the pathogen is controlled, the level of ROS in the insect decreases, leading to a decline in enzyme activities.

After feeding with *C. javanica*, the activities of POD and SOD in *S. frugiperda* reached their peak at 12 h post-treatment and then declined rapidly, whereas CAT activity remained consistently high throughout the experimental period. This may be attributed to the rapid accumulation of hydrogen peroxide following the increase in SOD activity within the gut. In a high-hydrogen-peroxide environment, POD activity rises quickly to prevent hydrogen peroxide from accumulating in cells. As the fungal infection progresses, the protective enzyme system of the insect is disrupted, leading to the suppression of SOD and POD expression. Meanwhile, CAT, as a key antioxidant enzyme responsible for scavenging H_2_O_2_, is independently upregulated when SOD and POD functions are compromised. It continues to function throughout the infection to cope with the sustained oxidative stress triggered by fungal invasion, thereby maintaining the basic redox balance of host cells. Consistent with our findings, feeding *M. anisopliae* to *Locusta migratoria manilensis* (Meyen, 1835) induced a similar trend in POD and SOD activities, with an initial increase followed by a decrease [78]. A similar response pattern for POD and SOD was also observed in *Tetranychus urticae* Koch, 1836 after feeding *Purpureocillium lilacinum* (Thom) Luangsa-ard, Houbraken, Hywel-Jones & Samson 2011. However, in contrast to our results, CAT activity in *T. urticae* was continuously inhibited following feeding, possibly due to pathogen-mediated suppression of arthropod CAT activity, which may compromise host immunity and facilitate further infection [79]. In another study, feeding *Akanthomyces attenuatus* (Zare & W. Gams) Spatafora, Kepler & B. Shrestha 2017 to *Megalurothrips usitatus* (Bagnall, 1913) exerted a sustained inhibitory effect on protective enzymes, ultimately leading to immune system collapse and host death [80]. In our study, the protective enzyme response to feeding treatment with BE01 was stronger than that to immersion treatment, suggesting that intestinal protective enzymes may be more actively mobilized in response to orally introduced pathogens.

The immersion treatment of *S. frugiperda* with *C. javanica* also induced alterations in the detoxification enzyme activities of the host. The activity of CarE initially decreased and subsequently increased, while GST exhibited a pattern of decrease–increase–decrease. This observed trend may be attributed to the progressive proliferation of the fungus within the insect, leading to an accumulation of harmful fungal secondary metabolites [81]. Consequently, the detoxification enzymes are activated to facilitate the clearance of these deleterious compounds [82]. In *Paralipsa gularis* (Zeller, 1877) treated with *Metarhizium rileyi* (Farl.) Kepler, S.A. Rehner & Humber 2014, the activities of CarE and GST showed a continuous increase, whereas in *Diaphorina citri* Kuwayama, 1908 treated with *Cordyceps fumosorosea* (Wize) Kepler, B. Shrestha & Spatafora 2017, the activities of both enzymes exhibited a sustained decrease [41,71]. These results indicate that the changes in insect detoxification enzyme activities vary depending on the entomopathogen involved. Unlike the results obtained from the immersion treatment, the activities of CarE and GST in *S. frugiperda* reached their peak at 12 h after feeding on *C. javanica*, and subsequently decreased continuously, yet remained consistently higher than those in the control group. This suggests that detoxification enzymes in the intestine play an immediate role during the early stage of fungal infection and continue to function thereafter. Similarly, the activities of CarE and GST in *T. cinnabarinus* following feeding with *P. lilacinum* exhibited comparable trends to those observed in our study [79]. *M. anisopliae* reduced CarE activity, suppressed the immune response of *S. frugiperda*, and resulted in larval mortality [56]. The GST activity in *Aphis craccivora* Koch, 1854 fed with the crude toxin of *Lecanicillium araneicola* Sukarno & Kurihara 2009 remained consistently higher than that of the control group, indicating a sustained detoxification effect [81]. Furthermore, our results indicated that the detoxification enzyme response to *C. javanica* immersion was stronger than that observed following feeding. This may be attributed to the higher activity of detoxification enzymes in the hemolymph in response to infection by entomopathogenic fungi.

Infection by *C. javanica* affects the expression of immune-related genes in *S. frugiperda*. In nature, entomopathogenic fungi primarily infect their hosts through direct penetration of the cuticle. Consequently, the insect’s immune defenses—particularly in the hemolymph—have evolved to mount effective responses against pathogens entering through this route. Consistent with this rationale, our immersion treatment (which simulates cuticular contact) revealed that the expression of *attacin A*, *lebocin 4*, *gloverin*, and *cecropin* in *S. frugiperda* was initially inhibited and subsequently up-regulated between 12 and 36 h after treatment, with expression levels exceeding those of the control group at 24 h and 36 h, peaking at 24 h. Subsequently, the expression of antimicrobial peptides (with the exception of *gloverin*) remained stable at 48 h and 60 h. We speculate that the humoral immunity of *S. frugiperda* was transiently suppressed during the very early stage of cuticular infection, possibly due to fungal evasion strategies, and subsequently activated to contain the pathogen. After the fungal infection was inhibited, the expression levels of antimicrobial peptides decreased and stabilized. Similarly, the expression of *attacin* in *Plagiodera versicolora* (Laicharting, 1781) at 12 h and 24 h after spraying with *B. bassiana* also exhibited a trend of initial inhibition followed by up-regulation [48]. In contrast to our findings, the expression levels of *attacin*, *lebocin 4*, *gloverin*, and *cecropin A* in *Ostrinia furnacalis* induced by injection with *P. aeruginosa* exhibited an initial increase followed by a decrease (with the exception of *lebocin 4*), peaking at 12 h post-infection [83]. It is possible that direct injection of pathogenic bacteria into the hemolymph bypasses the early fungal recognition and evasion phase, thereby triggering humoral immunity more rapidly than invasion of pathogenic fungi from the body surface. Notably, the feeding treatment (which bypasses the cuticular barrier and introduces conidia directly into the midgut) resulted in a markedly different immune response. Since the oral route is not the primary infection route in nature, the insect’s immune system may be less effectively triggered when the pathogen enters through this portal. Indeed, we found that feeding with *C. javanica* inhibited the immune response mediated by certain immune-related genes of *S. frugiperda*. The expression of *attacin A* (except at 36 h and 60 h) and *PGRP-LB* was inhibited at nearly all time points after feeding, which may be attributed to the down-regulation of *attacin* following the inhibition of *PGRP* [84]. Unlike *attacin* and *PGRP-LB*, the expression of *lebocin 4* was up-regulated at all time points except 24 h and 60 h, particularly at 36 h after feeding, exhibiting a 3007-fold increase. Similarly, *lebocin* expression was up-regulated in the fat body of silkworms following feeding with *E. coli* and *S. aureus* for 4 to 16 h [85]. However, *gloverin* exhibited a similar expression trend to that observed after immersion treatment (initial down-regulation followed by up-regulation), except that the peak occurred later than in the immersion treatment, reaching a level at 48 h that was 122 times that of the control group. Similarly, feeding *Galleria mellonella* larvae with *Bacillus thuringiensis* for 48 h resulted in more than a 30-fold up-regulation of *gloverin* expression in their midgut [86]. Taken together, these distinct immune responses between immersion and feeding treatments likely reflect the host’s evolutionary adaptation to recognize and contain fungal pathogens primarily via the cuticular route, whereas the oral route bypasses these first-line defenses and leads to altered immune outcomes.

## 5. Conclusions

In summary, feeding with *C. javanica* is more toxic to *S. frugiperda* than immersion treatment, resulting in higher mortality and a significantly lower pupation rate. However, neither feeding nor immersion treatment affected the female ratio of *S. frugiperda*. Moreover, feeding treatment induced more pronounced changes in protective enzymes and the expression of defense-related genes in *S. frugiperda* compared to immersion treatment. The results indicated that *S. frugiperda* must feed a contaminated substrate with the entomopathogenic fungi to be more efficiently infected. These results provide preliminary evidence for the potential of *C. javanica* BE01 as a biocontrol agent against *S. frugiperda*, although further studies are needed to elucidate the molecular mechanisms underlying the observed mortality and to evaluate its efficacy under field conditions. It should be noted, however, that only two concentrations were tested, and future studies with a broader concentration gradient are warranted to fully characterize the dose–response relationship.

## Figures and Tables

**Table 1:** Pupation rate and female proportion of *Spodoptera frugiperda* larvae immersed in different concentrations of *Cordyceps javanica*.

Bioassay	Treatment		Control	Number	χ^2^	*p*
	Conidia Suspension Concentration (Conidia/mL)		0.1% Tween 80 Sterile Water	*n*		
Pupation rate	1 × 10^4^	93.9%	93.9%	197	0.000	0.986
	1 × 10^6^	85.6%		195	3.658	0.056
Female proportion	1 × 10^4^	62.4%	58.7%	185	0.261	0.610
	1 × 10^6^	67.5%		175	1.439	0.230

**Table 2:** Pupation rate and female proportion of *Spodoptera frugiperda* larvae after feeding with different concentrations of *Cordyceps javanica*.

Bioassay	Treatment		Control	Number	χ^2^	*p*
	Conidia Suspension Concentration (Conidia/mL)		0.1% Tween 80 Sterile Water	*n*		
Pupation rate	1 × 10^4^	90.4%	96.5%	180	2.674	0.102
	1 × 10^6^	65.2%		152	25.900	<0.001 ***
Female proportion	1 × 10^4^	61.2%	62.7%	168	0.039	0.844
	1 × 10^6^	58.1%		126	0.243	0.622

***: Significant values corresponding to *p* < 0.001.

## Data Availability

The datasets presented in this study can be found in online repositories (https://www.ncbi.nlm.nih.gov/genbank/ (accessed on 30 March 2025)). The accession numbers for each dataset are provided in Table S1.

## Supplementary Materials

The following supporting information can be downloaded at: https://www.mdpi.com/article/10.3390/insects17090933/s1, Table S1: The primer for RT-qPCR of related genes of immunity from *S. frugiperda*; Table S2: Daily mortality counts of *S. frugiperda* larvae under immersion treatment; Table S3. Daily mortality counts of *S. frugiperda* larvae under feeding treatment.
