# Supplementary material for: Oral Exposure to Cordyceps javanica Reduces Survival and Pupation of Spodoptera frugiperda and Alters Enzyme Activities and Immune-Gene Expression
Source: Insects. 2026 Sep 6;17(9):933. doi: 10.3390/insects17090933 (PMC13607405; doi:10.3390/insects17090933)
Supplement: Supplementary file 1 [file insects-17-00933-s001.zip › insects-4487735-supplementary.pdf]

Table S1. The primer for RT-qPCR of related genes of immunity from *S. frugiperda*.

Gene name (Genbank)	5′-3′Forward primer	5′-3′ Reverse primer
lebocin-4-like (*lebocin 4*) (XM_050699397)	GTTCGTCCACATTTGAGA	ATGATAGATGGCAGCACC
gloverin-like (*gloverin*) (XM_050698505)	CAGAGGCAGTGGTATG	ATTCAGGCTTGTTATGT
attacin-A-like (*attacin A*) (XM_035585918)	GGGTTCGACGAAGATG	TCGGAGTTGAGGGTGA
Cecropin isoform X2 (*Cecropin*) (XM_050697897)	CTGAACCAAGGTGGAA	CTATGGCTGCTGCTGA
PGRP LB-like isoform X2 (*PGRP-LB*) (XM_035592753)	GGTGCTATCCTGTATTTCC	ATTCGTCTTCACTGGGTC
EF1α (U20139.1)	TGGGCGTCAACAAAATGGA	TCTCCGTGCCAGCCAGAAAT

Table S2. Daily mortality counts of *S. frugiperda* larvae under immersion treatment.

Days	Control (0.1% Tween 80 sterile water)	1×10^4 conidia/mL	1×10^6 conidia/mL
1			
2			
3	1		
4			
5	1		
6			
7			1
8			1
9		1	
10			
11			1
12			
13			
14			
15			
Total number of larvae	100	100	100
Total number of dead larvae	2	1	3

Table S3. Daily mortality counts of *S. frugiperda* larvae under feeding treatment.

**Days**	**Control (0.1% Tween 80 sterile water)**	**$1×10^4$ conidia/mL**	**$1×10^6$ conidia/mL**
1		1	
2	1	1	
3	1		
4	2		
5	3	2	1
6			
7	4	2	
8			
9			4
10			6
11			10
12			13
13	2		
14	1		
15			
Total number of larvae	100	100	100
Total number of dead larvae	14	6	34
